# Sulfurtransferases and Cystathionine Beta-Synthase Expression in Different Human Leukemia Cell Lines

**DOI:** 10.3390/biom12020148

**Published:** 2022-01-18

**Authors:** Halina Jurkowska, Maria Wróbel, Ewa Jasek-Gajda, Leszek Rydz

**Affiliations:** 1 Chair of Medical Biochemistry, Faculty of Medicine, Jagiellonian University Medical College, 7 Kopernika St., 31-034 Kraków, Poland; mtk.wrobel@uj.edu.pl (M.W.); leszek.rydz@uj.edu.pl (L.R.); 2 Department of Histology, Faculty of Medicine, Jagiellonian University Medical College, 7 Kopernika St., 31-034 Kraków, Poland; ewa.jasek@uj.edu.pl

**Keywords:** thiosulfate sulfurtransferase, 3-mercaptopyruvate sulfurtransferase, gamma-cystathionase, cystathionine beta-synthase, l-cysteine, leukemia cells

## Abstract

The studies concerned the expression of sulfurtransferases and cystathionine beta-synthase in six human leukemia cell lines: B cell acute lymphoblastic leukemia-B-ALL (REH cells), T cell acute lymphoblastic leukemia-T-ALL (DND-41 and MOLT-4 cells), acute myeloid leukemia—AML (MV4-11 and MOLM-14 cells), and chronic myeloid leukemia—CML (K562 cells). Reverse transcription-polymerase chain reaction (RT-PCR) and Western blot analysis were performed to determine the expression of thiosulfate sulfurtransferase, 3-mercaptopyruvate sulfurtransferase, gamma-cystathionase, and cystathionine beta-synthase on the mRNA and protein level. Interestingly, we found significant differences in the mRNA and protein levels of sulfurtransferases and cystathionine beta-synthase in the studied leukemia cells. The obtained results may contribute to elucidating the significance of the differences between the studied cells in the field of sulfur compound metabolism and finding new promising ways to inhibit the proliferation of various types of leukemic cells by modulating the activity of sulfurtransferases, cystathionine beta-synthase, and, consequently, the change of intracellular level of sulfane sulfur as well as H_2_S and reactive oxygen species production.

## 1. Introduction

**Figure 1:**
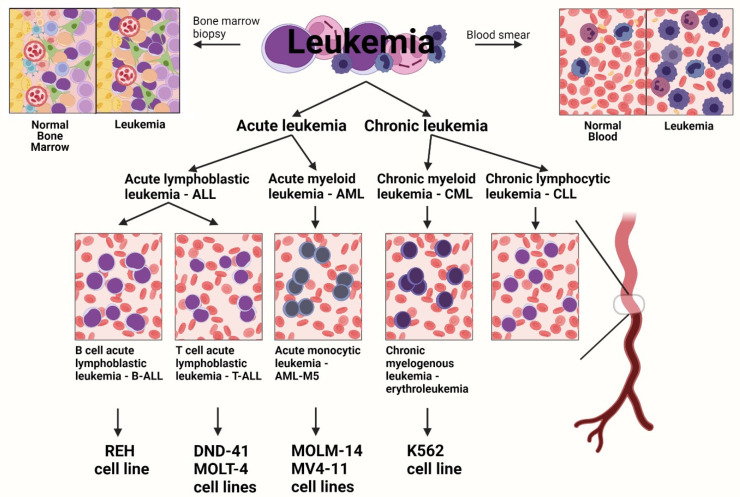
The different types of human leukemia cell lines. (Created with BioRender.com, accessed on 14 January 2022).

Leukemia is a type of cancer that affects the blood and bone marrow. Leukemia is classified by the cell phenotype (lymphocytic leukemia vs. myelogenous leukemia) and the rapidity of the clinical course (acute-fast-growing vs. chronic-slow-growing) (Figure 1). There are four major types of leukemia: acute lymphoblastic leukemia (ALL), acute myeloid leukemia (AML), chronic myeloid leukemia (CML), and chronic lymphocytic leukemia (CLL) [1,2].

Acute leukemias are malignant clonal disorders of the hematopoietic system. Acute leukemias are marked by the diffuse replacement of bone marrow with abnormal immature and undifferentiated hematopoietic cells, resulting in reduced numbers of erythrocytes and platelets in the peripheral blood [3].

Acute lymphoblastic leukemia—ALL—can be categorized as B-cell acute lymphocytic leukemia (B-ALL) and T-cell acute lymphocytic leukemia (T-ALL) [4].

Acute myeloid leukemia—AML (also known as acute myelogenous leukemia, acute myeloblastic leukemia, acute granulocytic leukemia, or acute nonlymphocytic leukemia)—is a highly aggressive and fast-growing form of malignancy of the stem cell precursors of the myeloid lineage (red blood cells, platelets, and white blood cells other than B and T cells) [5].

Acute lymphoblastic leukemia is typical of the pediatric age, whilst acute myeloid leukemia is more common in adult age [6].

Chronic leukemias encompass a broad spectrum of diseases characterized by uncontrolled proliferation and expansion of mature, differentiated cells of the hematopoietic system [3].

Chronic myeloid leukemia—CML (also known as chronic myelogenous leukemia)—belongs to the group of myeloproliferative neoplasms characterized by the uncontrolled growth of myeloid cells at different stages of maturation [7,8].

Chronic lymphocytic leukemia—CLL—is a malignant clonal proliferative disorder of mature CD5+ B lymphocytes that accumulate within the peripheral blood, lymphoid tissues, and bone marrow [9]. CLL, a cancer type with a p53-deficient subset, is one of the most common adult hematologic malignancies [9,10].

Our previous studies have shown that the proliferation of human cancer cells, such as astrocytoma (U373), glioblastoma (U87MG), and neuroblastoma (SH-SY5Y), depends on the expression/activity of sulfurtransferases and the level of sulfane sulfur [11,12,13,14]. Sulfurtransferases (thiosulfate sulfurtransferase, TST, rhodanese; 3-mercaptopyruvate sulfurtransferase, MPST; gamma-cystathionase, CTH) and cystathionine beta-synthase (CBS) are involved in l-cysteine metabolism [15]. These enzymes participate in: the formation of sulfane sulfur-containing compounds (TST, MPST, CTH) [16]; hydrogen sulfide (H_2_S) production (MPST, CTH, CBS) [17,18,19]; protection against oxidative stress (MPST, TST) [20,21]; mitochondrial bioenergetics (TST, MPST) [22,23]; glucose and lipid metabolism (MPST, TST) [24,25]; and cyanide detoxification (TST, MPST, CTH) [25,26]. Abe and Kimura [17] for the first time reported that H_2_S, which is produced in the brain largely by CBS, may also function as an endogenous neuromodulator.

In the present paper, we investigated the expression of TST, MPST, CTH, and CBS in the different types of human leukemia cell lines: B-ALL (REH cells), T-ALL (DND-41 and MOLT-4 cells), AML (MV4-11 and MOLM-14 cells), and CML (K562 cells).

## 2. Materials and Methods

### 2.1. Cell Culture

The studies were performed in human leukemia cell lines: REH (B cell acute lymphoblastic leukemia, B-ALL); DND-41 and MOLT-4 (T cell acute lymphoblastic leukemia, T-ALL); MOLM-14 and MV4-11 (acute myeloid leukemia, AML); and K562 (chronic myeloid leukemia, CML).

Human MOLT-4 and K562 cells were obtained from the European Collection of Cell Cultures (ECACC, Salisbury, UK). The human cell lines: REH, DND-41, MOLM-14, and MV4-11 were obtained from the German Collection of Microorganisms and Cell Cultures (DSMZ, Braunschweig, Germany).

All cell lines were cultured in RPMI-1640 GlutaMax medium supplemented with 10% fetal bovine serum (FBS) and 1% penicillin-streptomycin (100 Units/mL penicillin and 100 µg/mL streptomycin) (all reagents from Life Technologies, Carlsbad, CA, USA). The cells were maintained at 37 °C in a humidified 95% air and 5% CO_2_.

### 2.2. Isolation of Total RNA

Total cellular RNA was extracted from the leukemia cells using TRIzol reagent according to the manufacturer’s instructions (Invitrogen, CA, USA). Quantification and a purity assessment was performed using a NanoDrop ND-1000 Spectrophotometer (NanoDrop Technologies, Wilmington, DE, USA). The RNA integrity was judged by the clarity of ribosomal RNA bands visualized on agarose gel electrophoresis.

### 2.3. RT-PCR

The RT-PCR reaction mixture was performed as described previously [11]. Expressions of TST, MPST, CTH, CBS, and β-Actin genes were analyzed in leukemia cells.

For the TST gene, PCR cycling conditions were: 94 °C (5 min) for one cycle, 94 °C (30 s), 65.2 °C (30 s), and 72 °C (1 min) for 28 cycles, with a final extension at 72 °C (8 min). Primer sequences were as follows: forward 5′-CCA GCT GGT GGA TTC AAG GT-3′ and reverse 5′- CCC TTC TCG AAG CCA TCC TC-3′ (144 bp). The TST mRNA sequence was obtained from National Center for Biotechnology Information (NCBI). These PCR conditions for the TST gene are published for the first time in this paper.

For the MPST gene, PCR cycling conditions were: 94 °C for 5 min, 28 cycles of amplification (94 °C for 30 s, 56 °C for 30 s, and 72 °C for 2 min), and a final extension at 72 °C for 8 min. Primer sequences were as follows: forward 5′-CCA GGT ACC GTG AAC ATC CC-3′ and reverse 5′-TGT ACC ACT CCA CCC AGG A-3′ (227 bp) [11].

For the CTH gene, PCR cycling conditions were: 94 °C for 5 min, 28 cycles of amplification (94 °C for 30 s, 51 °C for 60 s, and 72 °C for 8 min), and a final extension at 72 °C for 10 min [27]. Primer sequences were as follows: forward 5′-GCA AGT GGC ATC TGA ATT TG-3′ and reverse 5′-CCC ATT ACA ACA TCA CTG TGG-3′ (301 bp) [28].

For the CBS gene, PCR cycling conditions were: 94 °C for 5 min, 38 cycles of amplification (94 °C for 30 s, 60 °C for 30 s, and 72 °C for 2 min), and a final extension at 72 °C for 8 min. Primer sequences were as follows: forward 5′-CGC TGC GTG GTC ATT CTG CC-3′ and reverse 5′-TCC CAG GAT TAC CCC CGC CT-3′ (300 bp) [29].

For the β-Actin gene, PCR cycling conditions were: 94 °C for 5 min, 28 cycles of amplification (94 °C for 30 s, 54 °C for 30 s, and 72 °C for 2 min), and a final extension at 72 °C for 8 min [27]. Primer sequences were as follows: forward 5′-CTG TCT GGC GGC ACC ACC AT-3′ and reverse 5′-GCA ACT AAG TCA TAG TCC GC-3′ (~300 bp) [30].

β-Actin was used as an internal standard to normalize all samples. The PCR amplified products were electrophoresed on 2.0% agarose gel containing ethidium bromide staining. The bands were visualized under UV light and photographed using the ChemiDocTM MP Imaging System (Bio-Rad, Hercules, CA, USA).

### 2.4. Western Blotting

Leukemia cells were lysed in buffer (50 mM Tris–HCl, pH 7.5, 150 mM NaCl, 1 mM EDTA, 0.5% NP-40) supplemented with 1X Complete Protease Inhibitor Cocktail (Sigma-Aldrich Corp., St. Louis, MO, USA). Cell lysates were centrifuged at 20,000× *g* for 15 min at 4 °C. Protein concentrations were determined using the bicinchoninic acid (BCA) assay (Thermo Scientific/Pierce Biotechnology, Rockford, IL, USA). Equal amounts of cell lysate (25 μg) were separated on a 12% SDS-polyacrylamide gel and then transferred to 0.22 μm PVDF membranes (Bio-Rad, Hercules, CA, USA). After blocking in 5% non-fat milk for 1 h, membranes were incubated in the primary antibodies overnight: anti-CBS (1:1000; mouse monoclonal, #H00000875-MO1, Abnova, Taiwan), anti-CTH (1:500; mouse monoclonal, #sc-374249, Santa Cruz Biotechnology, TX, USA), anti-MPST (1:800; rabbit polyclonal, #GTX108274, GeneTex, Inc., Irvine, CA, USA), anti-TST (1:800; mouse monoclonal, #66018-1-Ig, Proteintech Group, Rosemont, IL, USA), and anti-β-actin (1:1000; mouse monoclonal, #A1978, Sigma-Aldrich Corp., St. Louis, MO, USA). Goat anti-mouse alkaline phosphatase-conjugated antibodies (1:2000, Proteintech Group, Rosemont, IL, USA) and goat anti-rabbit alkaline phosphatase-conjugated antibodies (1:2000, Proteintech Group, Rosemont, IL, USA) were used as a secondary antibody. The immune complexes were visualized using nitro blue tetrazolium and 5-bromo-4-chloro-3-indolyl phosphate (NBT-BCIP) stock solution (Roche Applied Science, Penzberg, Germany). Densitometric analysis of proteins was performed using the ChemiDocTMMP Imaging System (Bio-Rad, Hercules, CA, USA). β-Actin was used as an internal control of the target proteins.

### 2.5. Statistical Analysis

Statistical analyses were performed using GraphPad Prism 9.0 (GraphPad Software Inc., La Jolla, CA, USA). The results were presented as the means ± standard deviations (SD). Each data set was analyzed by the Mann–Whitney test or Student’s *t*-test, with values of *p* < 0.05 as statistically significant. All the experiments were repeated at least three times.

## 3. Results

**Figure 2:**
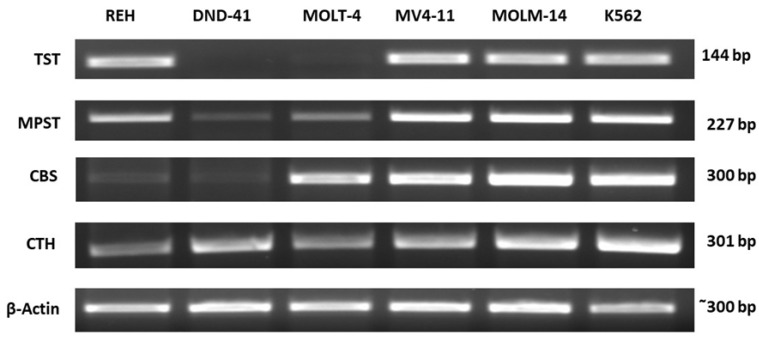
The expression of TST, MPST, CBS, and CTH on the mRNA level in leukemia cell lines (RT-PCR analysis). β-Actin was used as the loading control. Data from a representative experiment are presented. All the experiments were repeated at least three times with similar results.

**Figure 3:**
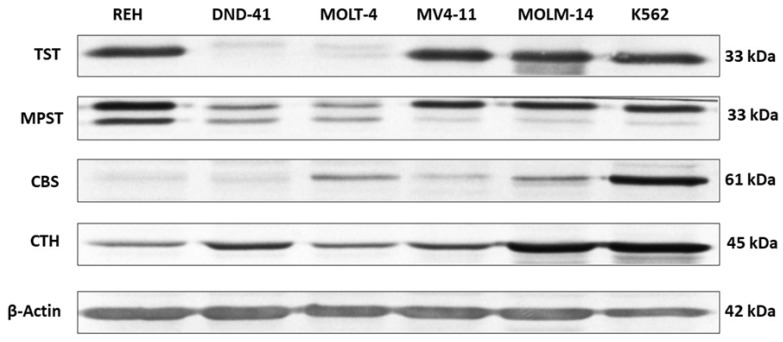
The expression of TST, MPST, CBS, and CTH on the protein level in leukemia cell lines (Western blot analysis). β-Actin was used as the loading control. Data from a representative experiment are presented. All the experiments were repeated at least three times with similar results.

In different types of leukemia cell lines, such as REH, DND-41, MOLT-4, MV4-11, MOLM-14, and K562, gene expression for TST, MPST, CBS, and CTH was studied on the mRNA and protein level. The representative results of these studies are shown in Figure 2 and Figure 3.

### 3.1. Thiosulfate Sulfurtransferase Expression in Leukemia Cell Lines

**Figure 4:**
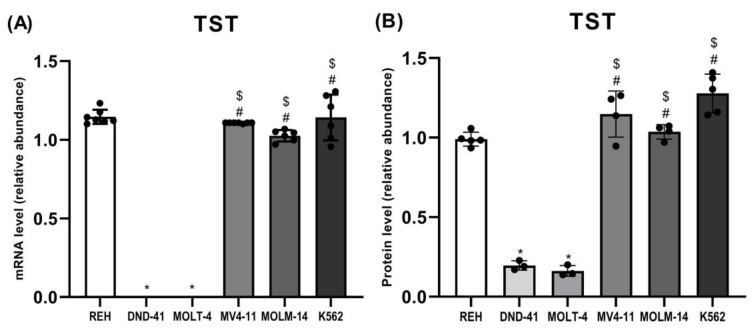
The expression on the mRNA (**A**) and protein (**B**) level of thiosulfate sulfurtransferase in leukemia cell lines (REH, DND-41, MOLT-4, MV4-11, MOLM-14, K562). β-Actin was used as a loading control. Quantification of the TST expression on mRNA level (RT-PCR method) and protein level (Western blotting method) was performed by densitometric analysis of the gels/blots and normalized to the internal loading control. The results are expressed as the mean ± SD of three or more independent experiments. Each mark represents *p* < 0.05 as followed: * versus REH; # versus DND-41; $ versus MOLT-4.

The results showed that the expression of TST (Figure 2, Figure 3 and Figure 4A,B) was comparable in REH, MV4-11, MOLM-14, and K562 cells. However, the expression of TST in DND-41 and MOLT-4 cells was negligible or not detected compared to the other studied cell lines.

### 3.2. 3-Mercaptopyruvate Sulfurtransferase Expression in Leukemia Cell Lines

**Figure 5:**
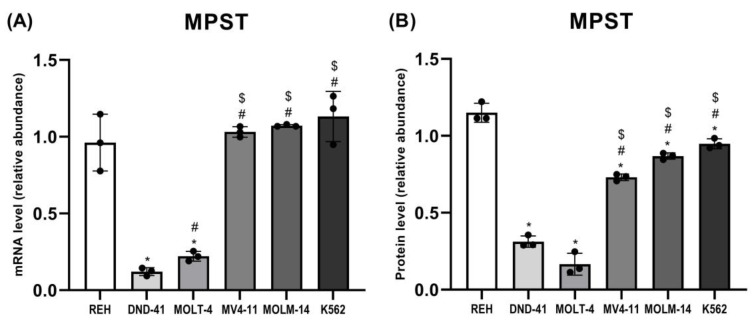
The expression on the mRNA (**A**) and protein (**B**) level of 3-mercaptopyruvate sulfurtransferase in leukemia cell lines (REH, DND-41, MOLT-4, MV4-11, MOLM-14, K562). β-Actin was used as a loading control. Quantification of the MPST expression on mRNA level (RT-PCR method) and protein level (Western blotting method) was performed by densitometric analysis of the gels/blots and normalized to the internal loading control. The results are expressed as the mean ± SD of three or more independent experiments. Each mark represents *p* < 0.05 as followed: * versus REH; # versus DND-41; $ versus MOLT-4.

We observed that the expression of MPST (Figure 2, Figure 3 and Figure 5A,B) in DND-41 and MOLT-4 cells was significantly lower as compared to the REH, MV4-11, MOLM-14, and K562 cells.

### 3.3. Cystathionine Beta-Synthase Expression in Leukemia Cell Lines

**Figure 6:**
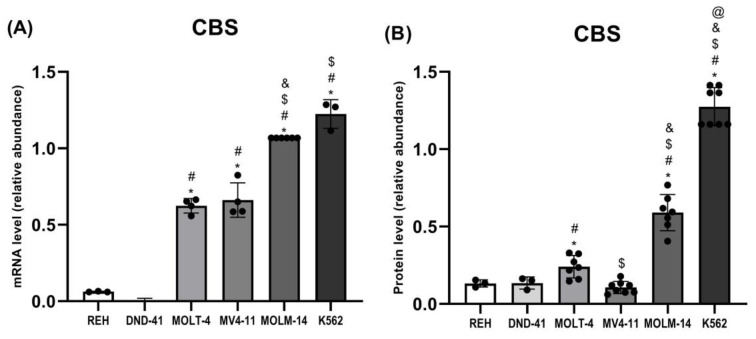
The expression on the mRNA (**A**) and protein (**B**) level of cystathionine β-synthase in leukemia cell lines (REH, DND-41, MOLT-4, MV4-11, MOLM-14, K562). β-Actin was used as a loading control. Quantification of the CBS expression on mRNA level (RT-PCR method) and protein level (Western blotting method) was performed by densitometric analysis of the gels/blots and normalized to the internal loading control. The results are expressed as the mean ± SD of three or more independent experiments. Each mark represents *p* < 0.05 as followed: * versus REH; # versus DND-41; $ versus MOLT-4; & versus MV4-11; @ versus MOLM-14.

Our analysis demonstrated that the expression of CBS (Figure 2, Figure 3 and Figure 6A,B) was the highest in MOLM-14 and K562 cells. In turn, the expression of CBS was significantly low in REH and DND-41 cells.

### 3.4. Gamma-Cystathionase Expression in Leukemia Cell Lines

**Figure 7:**
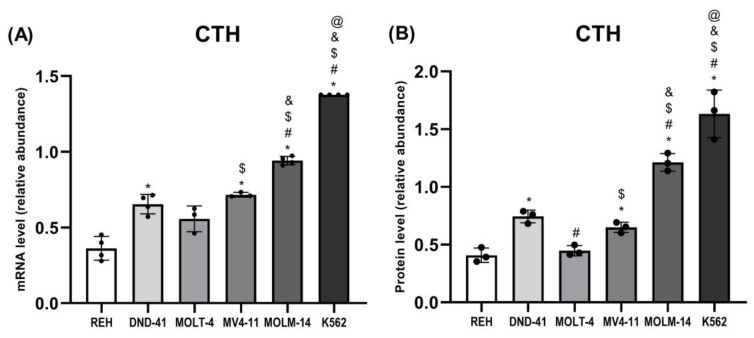
The expression on the mRNA (**A**) and protein (**B**) level of gamma-cystathionase in leukemia cell lines (REH, DND-41, MOLT-4, MV4-11, MOLM-14, K562). β-Actin was used as a loading control. Quantification of the CTH expression on mRNA level (RT-PCR method) and protein level (Western blotting method) was performed by densitometric analysis of the gels/blots and normalized to the internal loading control. The results are expressed as the mean ± SD of three or more independent experiments. Each mark represents *p* < 0.05 as followed: * versus REH; # versus DND-41; $ versus MOLT-4; & versus MV4-11; @ versus MOLM-14.

Our results also showed that the expression of CTH (Figure 2, Figure 3 and Figure 7A,B) was the highest in MOLM-14 and K562 cells as compared to the other studied leukemia cell lines.

## 4. Discussion

In this study, we examined the expression of three sulfurtransferases and cystathionine beta-synthase in different types of human leukemia cell lines. Interestingly, we found that in T-ALL cell lines, such as DND-41 and MOLT-4, the expression of TST (Figure 2, Figure 3 and Figure 4A,B) and MPST (Figure 2, Figure 3 and Figure 5A,B) were negligible. It is known that mitochondrial TST and MPST have antioxidant properties [20,21]. The catalytic site Cys247 of MPST (MPST–S-), which acts as a redox-sensing switch, is oxidized to MPST–SO-(cysteine sulfenate) and then reduced by reduced thioredoxin [20]. Moreover, Nandi et al. [31] demonstrated that one of the isoforms of TST acts as thioredoxin oxidase in vitro, suggesting a role of TST isoform in the detoxification of intramitochondrial reactive oxygen species (ROS). Wang et al. [32] reported that ROS levels were higher in T-ALL cells than in non-leukemic cells. Thus, the trace levels of TST and MPST in T-ALL cells (DND-41, MOLT-4) can additionally contribute to the high level of ROS in these cells.

It was found that the direct transport of mitochondria between cells can be used by cancer cells to sustain their high metabolic requirements and promote drug resistance. [33]. Tunneling nanotubes (ultrafine cytoplasmic bridges between cells) are the main delivery system for mitochondria from mesenchymal stem cells to primary B-cell precursor ALL cells [34] and T-cell ALL [32]. Mitochondria are exported from malignant T-ALL cells to the surrounding mesenchymal stem cells, probably due to the preferential use of glycolysis in T-ALL cells [32].

Peripheral T cell activation and proliferation are tightly controlled to prevent autoimmune destruction of cells [35]. T cells lack efficient transport of L-cysteine and are highly dependent on the extracellular availability of cystine imported through the cystine/glutamate antiporter xCT, which is expressed only after cell activation. Furthermore, T cells can import L-cysteine provided by antigen-presenting cells (macrophages, dendritic cells) through their ASC neutral amino acid transporter or thioredoxin-mediated reduction of cystine to L-cysteine [36]. T cell activation depends on hydrogen sulfide (H_2_S) signaling. H_2_S can act as an autocrine or paracrine T cell activator [37]. Moreover, this activation increases the capacity of T cells to H_2_S production via increased expression of CBS and CTH [38].

Interestingly, the results of the present study demonstrated that in B-ALL cells (REH) as well as in AML cells (MV4-11, MOLM-14) and CML cells (K562), the expression of mitochondrial TST (Figure 2, Figure 3 and Figure 4A,B) and MPST (Figure 2, Figure 3 and Figure 5A,B) was significantly higher as compared to T-ALL (DND-41, MOLT-4) cells.

Mitochondrial oxidative phosphorylation is enhanced in AML stem cells [39,40,41]. ALL cells export mitochondria to reduce intracellular ROS, while AML cells import mitochondria for the demand of oxidative phosphorylation [40]. Lagadinou et al. [39] showed that ROS-low AML stem cells, aberrantly overexpressing BCL-2, were unable to utilize glycolysis when mitochondrial respiration was inhibited; thus, the maintenance of mitochondrial function is essential for leukemia stem cell survival.

Mitochondrial sulfurtransferases, TST and MPST, participate in the formation of iron-sulfur (Fe-S) clusters [23]. Both enzymes, which are highly expressed in AML (MOLM-14, MV4-11) as well as B-ALL (REH) cells, may therefore play an important role in the formation of Fe-S clusters that are components of the respiratory chain complexes. Moreover, the high expression of mitochondrial TST in the studied MOLM-14, MV4-11, and REH cells can be involved in the oxidation of H_2_S to thiosulfate [22].

In this study, we showed that the expression of cytosolic CBS and CTH was the highest in MOLM-14 (AML) and K562 (CML) cells (Figure 2, Figure 3, Figure 6A,B and Figure 7A,B). Interestingly, in REH (B-ALL) cells, we have found low expression of CBS and CTH (Figure 2, Figure 3, Figure 6A,B and Figure 7A,B), but high expression of MPST and TST (Figure 2, Figure 3, Figure 4A,B and Figure 5A,B).

CBS and CTH are pyridoxal phosphate (PLP)-dependent enzymes [42]. CBS uses the PLP cofactor for the formation of L-cystathionine from serine and homocysteine in the transsulfuration pathway. CTH, in the presence of PLP, catalyzes the cleavage of L-cystathionine to L-cysteine, alpha-ketobutyrate, and ammonia. Both enzymes are also involved in H_2_S production [42]. CBS is upregulated in many types of cancer and also in leukemia cells [19]. Considering the results of our studies, it seems that CBS and CTH are the main enzymes involved in the production of H_2_S in AML (MOLM-14) and CML (K562) cells. We also conclude that MPST may have a significant impact on the formation of H_2_S in B-ALL (REH) cells.

Chen et al. [43] showed that vitamin B6 (PLP precursor) supports AML cell proliferation. AML cells are addicted to the vitamin B6 pathway, and its inhibition selectively impairs leukemic cell proliferation compared to other normal and cancer cell types. Recently, Wang et al. [44] reported that CBS expression was increased in the bone marrow mononuclear cells of pediatric CML patients, as well as in the CML-derived K562 cells, and CBS expression was correlated with different disease phases. Inhibition of CBS induced apoptosis and reduced cell proliferation in chronic myeloid leukemia [44].

In this research, we also showed a high expression of gamma-cystathionase in K562 cells (Figure 2, Figure 3 and Figure 7A,B). These results confirm the existing literature data, which shows the presence of CTH in chronic myeloid leukemia cells [45,46]. Glode et al. [45] reported that CTH was detected in K562 (CML) and HL-60 (AML) cell lines; these cell lines were not capable of sustained logarithmic growth in the absence of L-cysteine. However, cystathionine was capable of replacing the L-cysteine requirement for those lines [45].

It is known that sulfane sulfur donors can inhibit the proliferation of leukemic cells [47,48,49]. Diallyl disulfide (DADS, a sulfane sulfur donor isolated from garlic) decreased cell viability and increased apoptosis of myeloid leukemia cells (K562 and NB4) by inactivating the mTOR pathway [47]. DADS also induced apoptosis in HL-60 (AML) cells through the activation of NADPH oxidase and stimulation of ROS production [49]. Furthermore, GYY4137, a slow-releasing hydrogen sulfide donor, markedly reduced the growth and viability of HL-60 and MV4-11 cancer cell lines [48].

Our previous research has shown that in brain cancer and neuroblastoma cells, the increase in MPST activity by a sulfane sulfur donor (diallyl trisulfide) [11] and L-cysteine precursors (N-acetylcysteine, D-ribose-L-cysteine) [13,14] is associated with an increase in intracellular sulfane sulfur level and inhibition of proliferation of cancer cells. Thus, modulation of the activity of specific sulfurtransferases in different types of leukemic cells may change the intracellular level of sulfane sulfur and inhibit cell proliferation.

In conclusion, our findings showed significant differences in the expression of TST, MPST, CTH, and CBS in different types of human leukemia cells. The results of this study can contribute to finding appropriate ways to modulate the activity of sulfurtransferases as well as CBS and the level of sulfane sulfur and H_2_S in various types of leukemias, thereby regulating oxidative stress and inhibiting the proliferation of these cells.

## Data Availability

Not applicable.
